# The Effect of Gray Matter ICA and Coefficient of Variation Mapping of BOLD Data on the Detection of Functional Connectivity Changes in Alzheimer’s Disease and bvFTD

**DOI:** 10.3389/fnhum.2016.00680

**Published:** 2017-01-09

**Authors:** Timo Tuovinen, Riikka Rytty, Virpi Moilanen, Ahmed Abou Elseoud, Juha Veijola, Anne M. Remes, Vesa J. Kiviniemi

**Affiliations:** ^1^Department of Diagnostic Radiology, Oulu University HospitalOulu, Finland; ^2^Oulu Functional NeuroImaging group, Research Unit of Medical Imaging, Physics and Technology, the Faculty of Medicine, University of OuluOulu, Finland; ^3^Medical Research Center Oulu, Oulu University HospitalOulu, Finland; ^4^Research Unit of Clinical Neuroscience, Faculty of Medicine, University of OuluOulu, Finland; ^5^Department of Neurology, Institute of Clinical Medicine, University of Eastern FinlandKuopio, Finland; ^6^Department of Neurology, Kuopio University HospitalKuopio, Finland

**Keywords:** Alzheimer’s disease, behavioral variant frontotemporal dementia, coefficient of variation, gray matter, independent component analysis, quality control, resting-state fMRI

## Abstract

Resting-state fMRI results in neurodegenerative diseases have been somewhat conflicting. This may be due to complex partial volume effects of CSF in BOLD signal in patients with brain atrophy. To encounter this problem, we used a coefficient of variation (CV) map to highlight artifacts in the data, followed by analysis of gray matter voxels in order to minimize brain volume effects between groups. The effects of these measures were compared to whole brain ICA dual regression results in Alzheimer’s disease (AD) and behavioral variant frontotemporal dementia (bvFTD). 23 AD patients, 21 bvFTD patients and 25 healthy controls were included. The quality of the data was controlled by CV mapping. For detecting functional connectivity (FC) differences whole brain ICA (wbICA) and also segmented gray matter ICA (gmICA) followed by dual regression were conducted, both of which were performed both before and after data quality control. Decreased FC was detected in posterior DMN in the AD group and in the Salience network in the bvFTD group after combining CV quality control with gmICA. Before CV quality control, the decreased connectivity finding was not detectable in gmICA in neither of the groups. Same finding recurred when exclusion was based on randomization. The subjects excluded due to artifacts noticed in the CV maps had significantly lower temporal signal-to-noise ratio than the included subjects. Data quality measure CV is an effective tool in detecting artifacts from resting state analysis. CV reflects temporal dispersion of the BOLD signal stability and may thus be most helpful for spatial ICA, which has a blind spot in spatially correlating widespread artifacts. CV mapping in conjunction with gmICA yields results suiting previous findings both in AD and bvFTD.

## Introduction

Resting-state functional MRI (rs-fMRI) has been increasingly used in studies of neurodegenerative disorders in the recent years. It offers the benefit of the patient not having to be able to perform any specific tasks in the scanner and therefore it suits well in, e.g., dementia research. Alzheimer’s disease (AD) and behavioral variant frontotemporal dementia (bvFTD) are the two most common forms of early onset dementia. AD is typically associated with memory decline, but especially in early onset AD executive dysfunction and visuospatial dysfunction are also common (Mendez et al., 2007; Rohrer, 2012). bvFTD is characterized by profound changes in behavior and personality, as well as executive dysfunction (Rascovsky et al., 2011). Although the two disorders are anatomically and histopathologically distinct, considerable clinical overlapping exist, and the differential diagnosis may be difficult especially in the early stages of the disease. At present there are no reliable biomarkers and the diagnosis is based on clinical criteria.

In rs-fMRI studies the findings in AD have been quite consistent, and the finding of reduced default mode network (DMN) connectivity has been replicated in numerous studies (Zhou et al., 2010; Hafkemeijer et al., 2011; Agosta et al., 2012; Binnewijzend et al., 2012). However, in bvFTD the findings have been rather inconsistent. The finding of reduced salience network (SLN) connectivity has been reported most often, but the results have not been totally unanimous (Zhou et al., 2010; Farb et al., 2012; Filippi et al., 2012; Rytty et al., 2013; Lee et al., 2014). The inconsistency in the results may be related to the small study populations, varying fMRI data analyzing methods, different MRI field strengths, scanners and imaging sequences that have been used. Also the varying neuropathology and atrophy findings associated with bvFTD may have an impact. Other RSNs than the DMN and SLN have been studied only rarely in both disorders and the results have been heterogeneous (Farb et al., 2012; Filippi et al., 2012; Li et al., 2012; Rytty et al., 2013; Adriaanse et al., 2014; Lehmann et al., 2015).

The problem of the rs-fMRI signal is that it is noisy by nature and effective removal of artifacts has been gaining growing interest. Visual inspection of the data quality is important but that may not always be adequate. A recent study demonstrated that the finding of reduced functional connectivity (FC) in the DMN in AD could only be detected after aggressive data driven cleaning of the fMRI data using FMRIB’s ICA-based Xnoiseifier (FIX) (Griffanti et al., 2015). FIX attempts to auto-classify ICA components into RSNs and noise components. Noise components are regressed out of the 4D fMRI data before further analytics like dual regression. Reproducibility measurements of fMRI in resting state data indicate that ICA with dual regression is one of the most reliable fMRI metrics in light of reproducibility (Zuo and Xing, 2014). ICA can identify strongly independent noise sources that markedly alter signal probability distributions. However, if the artifacts and noise sources induce subtle alterations to the signal distributions in a way that they do not explain a lot of variance or if they are global in the image, their detection may be difficult even with ICA.

Coefficient of variation (CV) is a metric that is commonly used in e.g., engineering and analytical chemistry to measure quality and reproducibility. The metric is the ratio of the standard deviation to the mean and it reflects the dispersion of a frequency or probability distribution. As ICA algorithms utilize statistical properties of signal density distributions (Hyvarinen and Oja, 2000), the CV as a measure of dispersion of distributions sounds ideal for measuring noise quality in data intended for ICA analysis. Sudden movements (<< TR) like hiccup or cough during only part of the brain volume acquisition may induce signal intensity changes that will be missed by mere brain volume registration methods (Beall and Lowe, 2014). Also, technical gradient glitches during scanning may produce similar partial k-space alterations that are also hard to detect visually. In this article we utilize the CV mapping to detect these subtle technical signal changes that may be missed by either visual or motion parameter quality control. To our knowledge, CV has not been previously used in the context of fMRI quality control.

Brain atrophy has a known impact on measures of FC differences in neurodegenerative disorders (Lehmann et al., 2015). In our previous FC analysis we took gray matter atrophy into account by using gray matter as a spatial covariate in dual regression analysis of FC differences in bvFTD (Rytty et al., 2013, 2014). This may not be enough since loss of gray matter may induce for more of partial volume effects of CSF. Voxels with partial CSF contribution may alter connectivity measures as the CSF has markedly altered fluctuation pattern with high cardiovascular signal power (Kiviniemi et al., 2005, 2016; Bodurka et al., 2007). It has been shown that reducing partial volume effects improves measures of FC (Newton et al., 2012). Previously Formisano et al. (2004) have used cortex-based ICA focusing solely on gray matter. This method similarly improved the separation of cortical components and the estimation of their time courses particularly in the case of complex spatiotemporal statistical structure.

In this study we utilize CV mapping as a quality assurance metric and exclude subjects with CV images highlighting artifacts. We explore whether only a single 3D map of BOLD signal CV could be used in data quality control, thus speeding up the visualization process in addition to normal visual inspection of the whole 4D fMRI-data. Furthermore, we reduce the effects of gray matter loss by analyzing only voxels from gray matter on the individual level. We analyze the effect of CV quality control and gmICA on the FC changes on AD and bvFTD. We concentrate on the DMN in the AD group and on the SLN in the bvFTD group, which have been most widely studied in these disorders in previous research.

## Materials and Methods

**FIGURE 1:**
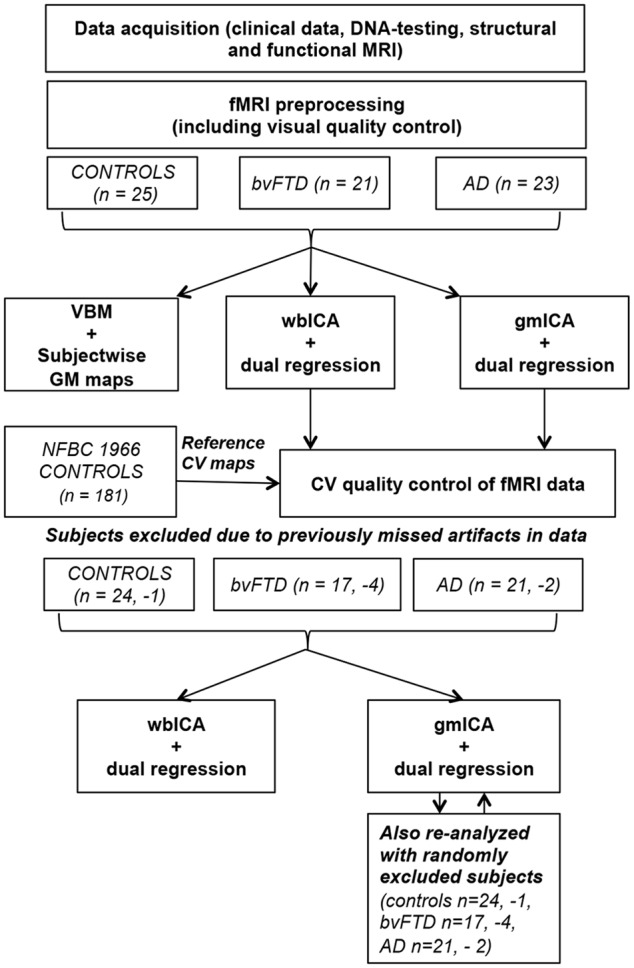
**A schematic summary of the study design**.

**Figure 1** provides a schematic summary of the study design.

### Participants

The study sample consisted of 23 patients with AD, 21 patients with bvFTD and 25 control subjects. All patients were examined in Oulu University Hospital at the Memory outpatient clinic of the Department of Neurology. The patients underwent a history and physical examination by a neurologist, thorough neuropsychological examination, screening laboratory tests and MRI. The neuropsychological examination was performed within 6 months of the fMRI scan of each patient. The controls were interviewed and MMSE and BDI were performed. No psychiatric or neurological disorders or medications affecting the central nervous system were allowed in the control group. Demographics and clinical data are summarized in **Table 1**.

**Table 1:** Subject demographics.

	AD (*n* = 23)	bvFTD (*n* = 21)	Controls (*n* = 25)	Overall ANOVA	AD/bvFTD (Mann–Whitney U)
F:M, n	14:9	10:11	13:12		
Age, years	61.5 (±5.6)	60.7 (±7.6)	59.6 (±5.3)	0.59	0.61
Disease duration, years	2.7 (±1.5)	3.0 (±1.8)	–		0.86
MMSE (max = 30)	22.3 (±3.0)	24.1 (±3.9)	28.9 (±1.1)	<0.001	0.08
FBI (max = 72)	NC	23.4 (±4.9) (*n* = 17)	NC		
BDI	NC	NC	3.1 (±3.3)		

*Values represent mean (SD). MMSE, Mini Mental State Examination; FBI, Frontal Behavioral Inventory Score; BDI, Beck’s Depression Inventory; NC, not collected.*

All patients in the AD group met the NINCDS-ADRDA (National Institute of Neurological and Communicative Disorders and Stroke and the Alzheimer’s Disease and Related Disorders Association) criteria for probable AD (McKhann et al., 1984). Cerebrospinal fluid measures were available from twelve AD patients and in all the cases they supported the diagnosis. Acetylcholinesterase inhibitors were used by 14, memantine by two and neuroleptic medication by four AD patients.

The bvFTD patients were clinically diagnosed according to the criteria of Lund and Manchester (Neary et al., 1998; Rascovsky et al., 2011). Patients presenting progressive aphasia and semantic dementia phenotypes were excluded. None of the patients had symptoms or signs suggesting amyotrophic lateral sclerosis. Medications for neuropsychiatric symptoms were used in some of the patients (acetylcholinesterase inhibitors in five patients, memantine in three, neuroleptics in eight and valproate in four). DNA samples were available from ten patients and seven of them carried the C9ORF72 repeat expansion (Renton et al., 2011). Mutations in progranulin or microtubule-associated protein tau genes were not found in any of the genetically tested patients.

Imaging data of 181 healthy subjects from Northern Finland Birth Cohort 1966 (NFBC 1966)^1^ was used to create normative CV maps (group mean and standard deviation).

Written informed consent was obtained from all of the patients or their legal guardians according to the Declaration of Helsinki. The Ethics Committee of the Northern Ostrobothnia Hospital District, Finland, approved all the research protocols.

### Image Acquisition

All subjects (including the NFBC participants) were imaged with a GE Signa HDx 1.5 T whole body system with an eight-channel receiver coil. Soft pads were fitted over the ears to protect hearing and to minimize motion. During MRI scanning all subjects received identical instructions: to simply rest and focus on a cross on an fMRI-dedicated screen, which they saw through the mirror system of the head coil.

#### Structural Imaging

High-resolution T1-weighted 3D FSPGR BRAVO images were taken in order to obtain anatomical images for co-registration of the fMRI data to the standard space coordinates and to investigate voxel-wise changes in the gray matter. Repetition time: 12.1 ms, echo time 5.2 ms, flip angle 20°, slice thickness 1.0 mm, field of view (FOV) 24.0 cm, matrix size 256 × 256 (i.e., 1 mm^3^ cubic voxels).

#### Functional Imaging

Resting-state BOLD data were acquired using a conventional gradient recalled echo (GRE) EPI sequence. Repetition time: 1800 ms, echo time 40 ms, 202 volumes, flip angle of 90°, 28 oblique axial slices, slice thickness 4 mm, inter-slice space 0.4 mm covering the whole brain, FOV 25.6 cm × 25.6 cm, matrix size: 64 × 64.

The TR was minimized in order to produce maximal number of volumes per scanning time, since ICA benefits from maximized number of volumes and statistical power. We optimized our protocol favoring high temporal resolution while still minimizing penalty on spatial resolution at 1.5 tesla system. The first three volumes were excluded from the time series due to T1 relaxation effects.

### Image Processing and Analysis

#### Analysis of Structural Imaging Data

Structural data were analyzed with FSL-VBM^2^, a voxel-based morphometry style analysis (Ashburner and Friston, 2000; Good et al., 2001). Structural images were brain-extracted using BET (Smith, 2002). This procedure was verified with visual inspection of the extraction result. Tissue-type segmentation into gray matter, white matter and CSF was carried out using FAST4 (Zhang et al., 2001). The resulting gray matter partial volume images were then aligned to Montreal Neurological Institute’s (MNI152) standard structural space template using the affine registration tool FLIRT (Jenkinson and Smith, 2001; Jenkinson et al., 2002), followed optionally by non-linear registration using FNIRT^3^, which uses a b-spline representation of the registration warp field (Rueckert et al., 1999). The resulting images were averaged to create a study-specific template, to which the native gray matter images were then non-linearly re-registered. The registered partial volume images were then modulated to correct for local expansion or contraction by dividing by the Jacobian of the warp field. The modulated segmented images were then smoothed with an isotropic Gaussian kernel with a sigma of 4 mm.

Finally, gray matter differences between different studies groups were statistically tested using permutation-based non-parametric testing incorporating threshold-free cluster enhancement (TFCE) (Smith et al., 2009). Resulting statistical maps were thresholded at *p* ≤ 0.05 (TFCE-corrected for family wise errors). The resulting subject-wise gray matter maps were also used in gray matter only ICA.

#### Functional Connectivity Analysis

The BOLD data were preprocessed with a typical FSL preprocessing pipeline as in our previous studies (Kiviniemi et al., 2009; Abou Elseoud et al., 2010). Head motion was corrected using MCFLIRT software (Jenkinson et al., 2002), and motion estimates computed by this algorithm were used in evaluating motion differences between groups.

Brain extraction was performed using FSL BET (Smith, 2002) with parameters *f* = 0.5 and *g* = 0; and for 3D FSPGR, *f* = 0.25 and *g* = 0. This procedure was verified with visual inspection of the extraction result. When the BET failed to satisfactorily remove some tissue, the extra cranial tissues (often in neck areas) were removed manually by removing the tissue with FSL and then re-entering the data into the processing pipeline. Smoothing as preprocessing step widens the spatial FC effects (Wu et al., 2011). In this paper we chose to minimize this effect. BOLD volumes were smoothed with only Gaussian kernel of 5 mm FWHM. Time series were high-pass filtered with an fslmaths tool using a 100-s cutoff. Multi-resolution affine co-registration within FSL 4.1.4 FLIRT software (Jenkinson et al., 2002) was used to co-register mean, non-smoothed fMRI volumes to 3D FSGR volumes of corresponding subjects, and to co-register 3D FSPGR volumes to the MNI152 standard space template. Both whole brain BOLD data and individually masked gray matter BOLD data was used for group ICA. The masking was based on the anatomical gray matter VBM masks (see above) that were then realigned to match the 4 mm voxel dimension of individual BOLD data.

Group ICA analysis was performed on whole brain (wbICA) and segmented gray matter only (gmICA) BOLD data using FSL 4.1.4 MELODIC software implementing probabilistic independent component analysis (PICA) (Beckmann and Smith, 2004). A multisession temporal concatenation tool in MELODIC was used to perform PICA related pre-processing and data conditioning in the group analysis setting. In this study ICA was performed separately to patient vs. control groups (AD vs. CON and bvFTD vs. CON) and different setups (wbICA and gmICA). Spatial ICA using 50 independent component maps (IC maps) was applied to detect RSNs from the study population of interest. The IC maps were thresholded using an alternative hypothesis test based on fitting a Gaussian/gamma mixture model to the distribution of voxel intensities within spatial maps and the probability of false negatives and false positives was set to equal relevance (*P* < 0.5) (Beckmann et al., 2005). ICs were identified as anatomically and functionally classical RSNs upon visual inspection by a neuroradiologist (VK) using previously described criteria (Kiviniemi et al., 2009; Smith et al., 2009). Salience and DMN networks were identified amongst RSNs as previously reported (Kiviniemi et al., 2009; Smith et al., 2009; Abou Elseoud et al., 2010).

The analysis for the differences between groups was carried out using an FSL dual regression technique that allows for voxel-wise comparisons of resting-state fMRI (Filippini et al., 2009; Littow et al., 2010; Veer et al., 2010; Abou Elseoud et al., 2011). This involves (A) using the group-ICA spatial maps in a linear model fit against the separate fMRI data sets, resulting in matrices (time-course matrices) describing the temporal dynamics for each component and subject, and (B) using these time-course matrices to estimate subject-specific spatial maps. The ICA template for the dual regression was selected from the group ICs. The dual regression analysis was performed with variance normalization (Allen et al., 2012). Statistical analysis using permutation testing (implemented in the FSL’s Randomize tool, 10,000 random permutations) was performed on the selected networks to obtain *p* < 0.05 significance at voxel level. Bonferroni correction was used to counter for multiple comparisons problem in gmICA analysis where two similar SLN IC’s were detected. The Juelich histological atlas incorporated in FSL and the Harvard-Oxford cortical and subcortical atlases (Harvard Center for Morphometric Analysis), which are provided with the FSL4 software were used to identify the anatomical characteristics of the resulting PICA maps. The FSL4 fslstats and fslmaths tools were used to calculate the number of non-zero voxels in the selected difference maps, and their t-score values.

#### Coefficient of Variation Maps

Mapping the CV in each voxel enables to assess the quality of the data using only single 3D volume in a single glance thus speeding up the visualization process in addition to normal visual inspection of the whole 4D fMRI-data. For each preprocessed fMRI dataset, a single subject CV map was calculated voxel-wise:

$$\begin{array}{c} CV_{map}=\frac{\sigma (X)}{\overset{-}{X}}, \end{array}$$

where σ is standard deviation, X is voxel timeserie and 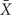 is mean voxel time serie.

**FIGURE 2:**
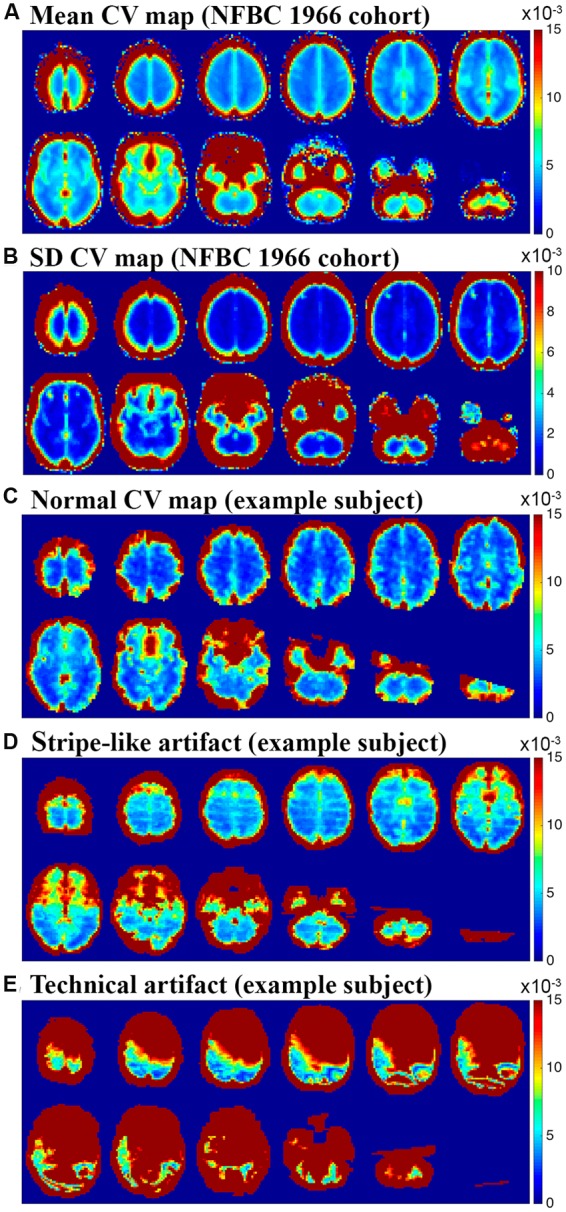
**(A,B)** Mean and standard deviation CV maps obtained from 181 healthy subjects from NFBC 1966. These maps were used as a reference to detect artifacts. **(C)** Example of single subject CV map considered normal **(D)** shows stripe-like artifact along slice orientation that was found in 7 subjects who were removed from the final analysis due to this artifact **(E)** shows another technical signal level artifact that was found from two subjects who were removed from the final analysis.

Data for reference CV map was obtained from 181 subjects from NFBC 1966 study. These single subject CV maps were merged into normative group mean and standard deviation CV maps using fslmaths. The group mean CV map was used as a visual reference for discarding artifactual data. **Figure 2** shows examples of excluded subject data with marked artifacts. Only clear visual aberration was considered significant.

#### The Effect of Subject Exclusion

The effect of excluding subjects from each study group based on CV quality control was tested. The original gmICA analysis was re-analyzed (a new group ICA and dual regression with 10,000 random permutations; 25 control subjects, 21 bvFTD patients and 23 AD patients), this time excluding randomly selected subjects (-1 control subject, -4 bvFTD patients and -2 AD patients) without considering the CV findings. This obviously is not exhaustive testing, but multiple group ICA and dual regression was not considered computationally feasible.

The temporal signal-to-noise ratio (tSNR) was evaluated. SNR is a measure that compares the level of a signal to the level of background noise. tSNR is defined as (Triantafyllou et al., 2005):

$$\begin{array}{c} tSNR=\frac{\overset{-}{X}}{\;\sigma (X)}, \end{array}$$

where σ is standard deviation, X is voxel timeserie and 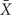 is mean voxel time serie.

The mean tSNR was calculated using voxels within MNI52 4 mm brain mask. The effect of gmICA was also evaluated comparing these mean tSNRs to the ones calculated using individually formed GM maps. Statistical testing was carried out using Mann–Whitney *U*-test.

## Results

### Coefficient of Variation Maps Highlight the Artifacts

For most of the subjects CV maps looked consistent and no technical artifacts were detected by visual inspection (**Figure 2C**). For seven subjects (1 control subject, 4 bvFTD patients and 2 AD patients), CV maps revealed stripe-like slice direction artifacts that were not clearly visible in the pre-processing stage (**Figure 2D**). These artifacts could not be easily detected in visual re-evaluation and not in the MCFLIRT motion parameter of the 4D fMRI-data. Two subjects in the AD group showed clear, widespread signal defect artifacts (**Figure 2E**). These artifacts were not detected in the preprocessing stage, but in visual re-evaluation of the 4D fMRI-data these subtle artifacts could now be clearly detected.

### Motion

There were no significant differences in the head motion parameters in the absolute [AD (0,25 mm), bvFTD (0.25 mm), CON (0,23 mm), *p* > 0.05] or relative [AD (0,08 mm), bvFTD (0.07 mm), CON (0,06 mm), *p* > 0.05] head motion between the study groups. Maximum absolute (0.96 mm) and relative (0.15 mm) head motion were below the voxel size in all subjects.

### Structural Findings in AD and bvFTD

In AD most prominent atrophy was detected in precuneus and posterior cingulate gyrus. Significant atrophy was also detected in bilateral angular gyri, left precentral gyrus and bilaterally in temporal lobes and hippocampi.

**FIGURE 3:**
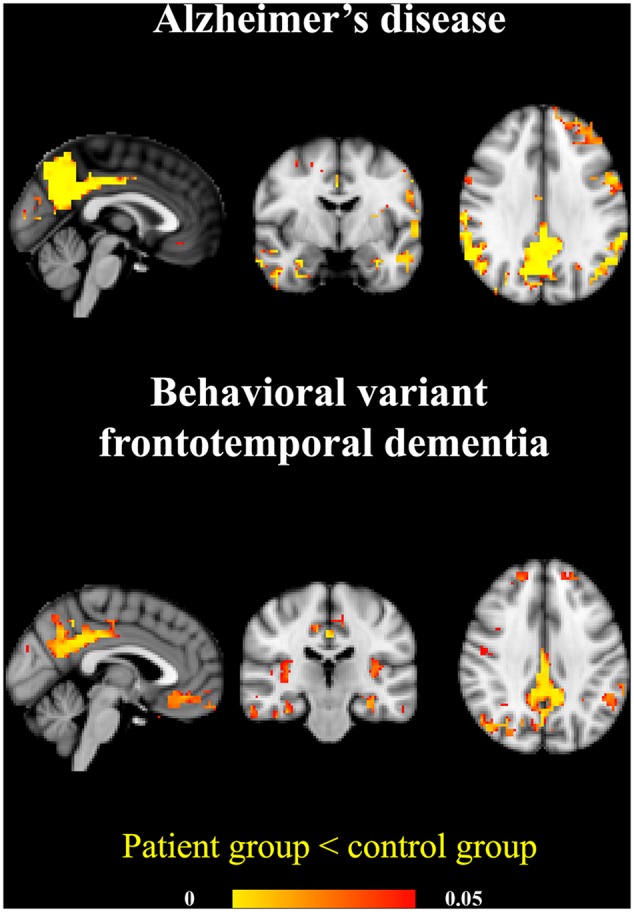
**Atrophy patterns in AD and bvFTD.** The AD group showed prominent temporoparietal atrophy. In the bvFTD group the atrophy was located in posterior cingulate gyrus and precuneus and also on frontal lobes and insula. Significant atrophy is marked in yellow (*p* < 0.05).

In bvFTD atrophy was detected in posterior cingulate gyrus and milder atrophy was also detected in precuneus. Additionally, more widespread atrophy was detected in frontal medial cortex, inferior temporal gyrus, central opercular and insular cortices and left hippocampus. The structural findings are displayed in **Figure 3** and **Table 2**.

**Table 2:** Statistics of significant differences in gray matter anatomical volume.

			Coordinates			*t*-score			
	Voxels	Volume	*X*	*Y*	*Z*	Mean	Std	Min	Max
AD	3614	231296	30	25	12	2.78	0.75	1.85	7.39
bvFTD	1732	110848	31	16	30	3.00	0.59	2.16	6.17

*Results are demonstrated by the number of voxels (4 mm), volume in mm^*3*^, mean, standard deviation, minimum and maximum of the *t*-scores. MNI coordinates of the maximum change of the involved anatomical areas.*

### Functional Connectivity Findings before CV Quality Control

#### Whole Brain ICA – **Figure 4A**, **Table 3**

**FIGURE 4:**
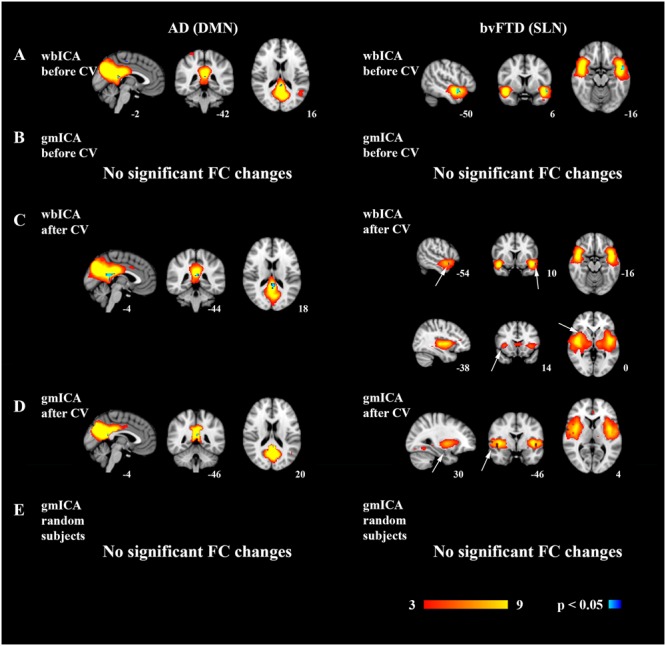
**Reduced functional connectivity changes in the AD and bvFTD groups.** In AD, the default mode network (DMN) and in bvFTD the salience network (SLN) was analyzed. Group ICA and dual regression were carried out for the whole brain, i.e., for all the voxels (wbICA) or for gray matter only (gmICA), and the network of interest was recognized from the group ICA results. Decreased connectivity was found in the DMN in AD and in the SLN in the bvFTD group in the wbICA **(A)**. However, the results were not statistically significant in the gmICA **(B)**. After CV quality control, decreased FC was detected in the DMN in AD and in the SLN in bvFTD in wbICA **(C)** and importantly also in gmICA **(D)**. A new gmICA analysis was performed after returning the subjects originally excluded based on CV quality control and excluding the same amount of randomly selected subjects. No significant FC changes were detected **(E).**

**Table 3:** Decreased functional connectivity detected in the DMN in AD patients and in the SLN in bvFTD patients when compared to healthy controls.

				Coordinates			*t*-score			
		Voxels	Volume	*X*	*Y*	*Z*	Mean	Std	Min	Max
AD	**DMN**									
	wbICA non-CV	73	4672	23	21	22	3.20	0.49	2.48	5.05
	wbICA CV	22	1408	23	21	22	3.82	0.44	3.31	4.98
	gmICA non-CV	^∗^								
	gmICA CV	5	320	23	20	23	4.54	0.37	4.09	4.94
bvFTD	**SLN**									
	wbICA non-CV	13	832	35	32	16	4.19	0.47	3.62	5.07
	wbICA CV	86	5504	22	7	19	3.44	0.52	2.70	5.15
	wbICA CV	9	576	35	32	16	4.05	0.34	3.72	4.64
	gmICA non-CV	^∗^								
	gmICA CV	5	320	8	31	16	4.05	0.37	3.72	4.68

*The results of the wbICA and gmICA analysis both with and without CV quality control are displayed. Two SLN components showed decreased connectivity in the bvFTD group in wbICA followed by CV. ^∗^In the gmICA without CV quality control, no changes were detected in neither of the patient groups. Significant differences are demonstrated by the number of voxels (4 mm), volume in mm^*3*^, mean, standard deviation, minimum and maximum of the *t*-scores of randomize tstat-files. MNI coordinates of the maximum change of the involved anatomical areas.*

In wbICA followed by dual regression, the AD group showed decreased connectivity in posterior cingulate gyrus in the posterior DMN. In the bvFTD group decreased FC was detected in the left insula, which is part of the SLN.

#### Gray Matter Only ICA – **Figure 4B**

gmICA followed by dual regression was conducted in order to account for gray matter atrophy. When the artifacts were not efficiently removed from the data, no changes were detected in the DMN in the AD group or in the SLN in the bvFTD group.

### Functional Connectivity Findings after CV Quality Control

#### Whole Brain ICA – **Figure 4C**

After removal of artifacts based on CV quality control, wbICA and dual regression were conducted again in both patient groups. Significant FC reductions were detected in both groups. In AD, reduced FC was seen in the posterior DMN. In bvFTD, reduced FC was seen in separate bilateral insular salience components even after Bonferroni correction for multiple comparisons.

#### Gray Matter Only ICA – **Figure 4D**

After CV quality control, gmICA was conducted in order to account for disease-related atrophy. After concentrating solely on gray matter, the detected FC differences in the DMN in AD and in the SLN in bvFTD were smaller than before atrophy correction. Nevertheless, after effective removal of artifacts, the AD group still showed significantly decreased FC in the precuneus in the DMN. In the bvFTD group, reduced FC was seen in the right insula in the SLN. **Table 3** shows statistics of the changes in the FC of the areas.

#### The Effect of Subject Exclusion – **Figure 4E**

A new gmICA was performed to test the effect of reduced size of study groups due to the CV quality control. The subjects originally excluded from the study based on CV quality control were returned and the same amount of different subjects was randomly excluded. With these novel study groups gmICA followed by dual regression was conducted. No statistically significant changes were detected in the DMN in the AD group or in the SLN in the bvFTD group.

### Temporal Signal-to-Noise Ratio

**FIGURE 5:**
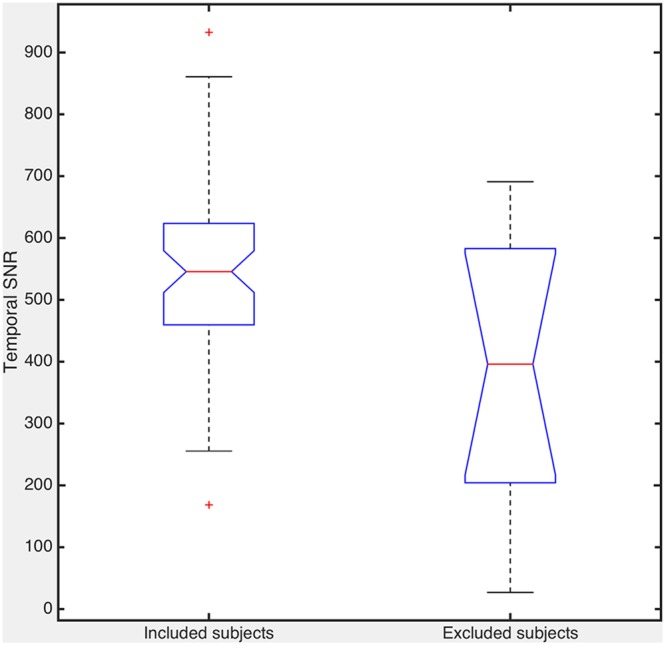
**The mean temporal signal-to-noise ratio (tSNR) comparing the subjects included and excluded based on the CV quality control.** The difference between these two groups is statistically significant (*p* = 0.0237).

The subjects excluded due to artifacts noticed in CV maps had significantly lower tSNR than the included subjects (*p* = 0.0237, c.f. **Figure 5**). Gray matter template did not have statistically significant effect to the mean tSNR of the signal (*p* = 0.955).

## Discussion

In this study FC changes in AD and bvFTD were analyzed using two simple approaches of controlling data variance. The overall data quality of the fMRI signal was first evaluated by calculating CV maps, a novel quality control method introduced in this paper. This method revealed artifacts in the data missed in the original visual inspection and other preprocessing stages such as motion control. Based on CV findings, some patients were removed from the final analysis in both patient groups. We show that this additional data quality control is helpful in clinical cases.

Secondly, we reduce unnecessary data variance of the clinical BOLD datasets by focusing the analysis on gray matter. Furthermore, the considerable gray matter atrophy in neurodegenerative diseases like AD and bvFTD needs to be addressed in order to minimize false positive changes in FC. In the wbICA, the effects of atrophy are not accounted for and we therefore performed ICA with gray matter only analysis. In this analysis with strict atrophy correction, the finding of reduced connectivity in the DMN in AD and in insula (part of the SLN) in bvFTD could only be detected when the subjects showing artifacts on their CV maps were removed from the analysis. The effect of excluding subjects was tested by re-analyzing the gmICA, but this time including those with artifacts within the BOLD data and excluding random subjects. With this approach the reduced connectivity was not detected. In our opinion, this highlights the importance of quality control in the fMRI data. In this study, our focus was to improve detection of artifacts and we did not attempt to clean the data but methods exist for cleaning procedures as well.

To detect artifacts in the BOLD data we used CV maps formed individually for each subject. CV addresses dispersion of probability and frequency distributions and as such suits for a quality measure for data attributed to ICA. The ICA utilizes the skewedness, kurtosis or other higher order statistics, i.e., the shape of the signal source joined density distributions in separating non-Gaussian, un-correlated signal sources (Hyvarinen and Oja, 2000). Probabilistic ICA utilizes gamma distributions fitted to distribution tails (Beckmann and Smith, 2004). If the data has some odd dispersion in the signal distributions, like widely spatially correlated sudden signal changes affecting only parts of the k-space, those may mask brain activity sources and therefore affect subtle group differences as well. In order to obtain normative mean and standard deviation CV maps we used imaging data of 181 healthy subjects from NFBC 1966 scanned with the same scanner with identical imaging parameters. Based on the normative data from this large sample we were able to use it as a reference to detect spatially widespread technical or motion related signal changes as shown in **Figures 2D,E**. These data alterations may be hard to detect even with spatial ICA since some ICA algorithms tend to detect spatially sparse events (Daubechies et al., 2009). The temporal signal-to-noise ratio of the removed subjects was also lower. Therefore, an additional quality measure such as CV mapping does seem to improve the accuracy for subtle changes between groups in the data.

Altogether, our FC results after CV quality control and gray matter only ICA are in line with previous literature. In this study, decreased FC was seen in the precuneus in the DMN in the AD group. The finding of reduced posterior DMN connectivity has been widely replicated in AD (Zhou et al., 2010; Hafkemeijer et al., 2011; Agosta et al., 2012; Binnewijzend et al., 2012). Decreased FC in the right insula was found in the bvFTD group. The insula is part of the salience network, which has been associated with guiding of behavior (Seeley et al., 2007; Pievani et al., 2014). The finding of reduced FC in the salience network has been replicated in most rs-fMRI studies concerning bvFTD (Zhou et al., 2010; Whitwell et al., 2011; Borroni et al., 2012; Farb et al., 2012; Filippi et al., 2012).

The detected atrophy in AD and bvFTD groups in this study is consistent with previous literature (Du et al., 2007; Tartaglia et al., 2011; Hartikainen et al., 2012; Whitwell and Josephs, 2012). The finding of posterior atrophy in the bvFTD group may be at least partly driven by the patients with the C9ORF72 expansion, since it has been associated with more widespread cortical atrophy involving also the parietal lobes and the cerebellum (Boeve et al., 2012; Whitwell et al., 2012). The significant atrophy that is present in neurodegenerative diseases has to be accounted for in the FC analysis. At present, the ideal means for consideration of atrophy is still under investigation.

In previous studies concerning bvFTD or AD various methods for atrophy correction have been used, which may have an impact on the results. Often gray matter loss has been used as a covariate in the FC analysis (Zhou et al., 2010; Binnewijzend et al., 2012). In this study we used a strict atrophy correction method and only concentrated on gray matter in ICA in order to minimize the CSF partial volume effect and to increase sensitivity to BOLD signal changes. The removal of white matter and CSF containing voxels in the gmICA also minimizes the effects of spurious CSF fluctuations that also affect connectivity measures (Bodurka et al., 2007).

Although the patient groups in this study were comparable in size with other fMRI-studies in neurodegenerative disorders, they are still rather small. After CV quality control the patient groups were still reduced and this reduction in power may have an effect on our results. To evaluate this we repeated the gmICA analysis including those originally excluded by the CV quality control and excluding randomly selected subjects. Results showed no significant FC changes in the RSNs studied. This implies that the results are not depending on the number of subjects but rather on the removal of clear artifacts showed in the CV maps.

Since the early differential diagnosis of AD and bvFTD is difficult, it is possible that some patients with atypical AD are placed in the bvFTD group and vice versa. Nevertheless, the follow-up time of the patients has been relatively long and they all underwent extensive diagnostic screening fulfilling the diagnostic criteria. In seven bvFTD patients genetic testing confirms the diagnosis. Unfortunately, there is no neuropathological confirmation of the diagnosis in any of the patients.

Expectations for rs-fMRI as a diagnostic biomarker in neurodegenerative diseases are high. However, at present significant results are achieved only on group level analysis and single-subject analysis are still under development. The quality control of the data is essential especially on single-subject level, which is mandatory in clinical work. CV maps introduced in this study are calculated on single subject level and seem to enable improved detection of artifacts.

### Comparison of Different Analytical Approaches

The classical whole brain analysis without correction with CV maps yields largest changes overall in the brain. After removing of the datasets with technically distorted CV maps, the volumes of the changes reduce in AD but increase in bvFTD. The wbICA results tend to have abnormal spurious connectivity changes outside the main RSN, like wbICA CV map corrected results in bvFTD finding maximum change in connectivity near sagittal sinus (MNI 22,7,19-coordinates). To counter the spurious FC alterations, the analyses were performed to data containing only gray matter. Without CV map correction of technically flawed data, the results failed to produce any significant FC changes. When the subjects with technical CV map distortions were removed, the gmICA results showed overlapping FC changes with the original areas but the alterations were focused solely within the RSN areas without spurious long distance abnormalities far from the proper network.

### Conclusion

In this study we highlight the meaning of quality control in rs-fMRI. We performed CV quality control to reveal artifacts and concentrated only on gray matter in the ICA in order to account for disease-related atrophy. With this approach, we detected decreased FC changes in the DMN in the AD group and in the SLN in the bvFTD group.

## Author Contributions

TT, RR, AR, and VK designed the study; collected, analyzed and interpreted the data; drafted and revised the manuscript; gave final approval. VM, AAE, and JV collected, analyzed, and interpreted the data; revised the manuscript; gave final approval.

## Conflict of Interest Statement

The authors declare that the research was conducted in the absence of any commercial or financial relationships that could be construed as a potential conflict of interest.

## Footnotes

1 www.oulu.fi/nfbc

2 www.fmrib.ox.ac.uk/fsl

3 www.fmrib.ox.ac.uk/analysis/techrep
